# Rate of Drug Coating Dissolution Determines In-Tissue Drug Retention and Durability of Biological Efficacy

**DOI:** 10.1155/2019/9560592

**Published:** 2019-01-08

**Authors:** Juan F. Granada, Renu Virmani, Daniel Schulz-Jander, Stefan Tunev, Robert J. Melder

**Affiliations:** ^1^CRF Skirball Center for Innovation, Columbia University, USA; ^2^CVPath Inc., Gaithersburg, MD, USA; ^3^Medtronic PLC, Cardiovascular Group, Santa Rosa, CA 95403, USA

## Abstract

Two different drug-coated balloons (DCBs) possessing different coating formulations were compared for rate of coating dissolution* in vitro,* in addition to tissue drug concentration and histological responses of treated vascular tissue* in vivo, *to determine if the rate of drug bioavailability to vascular tissue can impact the degree and duration of the observed pharmacological response to locally delivered drug.* In vitro* dissolution comparison demonstrated that a urea/paclitaxel-based coating formulation (IN.PACT™ Admiral™) released drug from solid to soluble phase at a slower and constant rate, yielding approximately 7% solubilized drug in 24 h. In contrast, a coating formulated from polysorbate/sorbitol/paclitaxel (Lutonix™) released 51% of solid phase drug to soluble phase in 1 h of dissolution with the remainder solubilizing in 24 h.* In vivo* evaluation of tissue drug concentration of both products showed significantly different tissue pharmacokinetic profile, with a higher concentration of paclitaxel in tissue at 90 days with a urea-based formulation excipient. Histological comparison of smooth muscle cell loss in response to drug exposure revealed contrasting trends of smooth muscle cell loss from 28 to 90 days with significantly higher response to drug observed at 90 days with the urea-based formulation. Rapid dissolution of drug from the polysorbate/sorbitol coating formulation was associated with an early increase in local cellular response to drug which diminished over 90 days with clearance of local drug from tissue. Sustained long-term drug-in-tissue concentration associated with the urea-based formulation demonstrated sustained pharmacological activity at 90 days, suggesting that slow coating dissolution provides a sustainable long-term tissue response.

## 1. Introduction

Through continued innovation, percutaneous treatment of coronary and peripheral stenosis has evolved rapidly since balloon angioplasty was first introduced three decades ago. Significant advances were made with the introduction of bare metal stents and subsequently drug-eluting stents, which expanded the possibility of successful revascularization in complicated lesions. Despite these advantages, efforts are still ongoing to improve patient outcomes further. In recent years, drug-coated balloons (DCBs) have emerged as a promising technology capable of providing a durable antirestenotic response but leaving no permanent implants behind. In the coronary territory, DCBs have proven to be efficacious in complex subsets such as in-stent restenosis, small vessels, and diffuse lesions where stent results are suboptimal. In contrast, in the peripheral vascular field, DCBs are becoming first-line of therapy. Several DCBs developed for coronary and peripheral applications have been extensively evaluated in preclinical and clinical studies with encouraging results [1] and have established its role in percutaneous vascular intervention [2]. However, there remain questions regarding the influence of drug formulation differences on tissue pharmacokinetics and the durability of the vascular response. This study was conducted to examine the effect of drug solubilization rate on the durability of drug in tissue and the resulting long-term effect on medial smooth muscle cells (SMC) in the treated artery.

Paclitaxel, the active pharmaceutical ingredient (API) coated on the IN.PACT Admiral DCB and Lutonix 0.35 DCB, is a lipophilic, poorly water soluble compound [3]. The activity of the drug is dependent upon solubilization by plasma matrix components and transport into the tissue. The purpose of this study was to investigate the rate at which two different paclitaxel-based coating formulations dissolve into the biologically relevant media and compare this property with resulting* in vivo* tissue pharmacokinetics and histological responses of the treated vascular tissue.

## 2. Materials and Methods

### 2.1. In Vitro Dissolution Analysis

Six IN.PACT Admiral DCBs (Medtronic PLC, Minneapolis, MN, USA; 6x80 mm) and six Lutonix DCBs (Bard, New Hope, MN, USA; 5x100 mm) were submerged into tubes containing 65 mL of porcine plasma at room temperature and deployed with gentile agitation for 20 seconds to dislodge the adherent coating. The balloons were then removed from the tubes and the tubes were placed horizontally in a 37°C shaking air bath at 50rpm. For the IN.PACT Admiral samples, aliquots of 0.5-mL were taken at 1, 2, 4, 6, and 24 hours. In addition to the sampling timepoints used for IN.PACT Admiral, the Lutonix DCB also required two earlier time points, at 0.167 hr (10 min) and 0.5 hr, in order to accommodate a faster elution profile without loss of early rate data. All aliquots were filtered with a 5-*μ*m filter to remove undissolved paclitaxel and then stored in a −80°C freezer until liquid chromatography-tandem mass spectrometry (LCMSMS) analysis (Agilent 1290 Infinity LC series, Santa Clara, CA, USA with AB SciEx 4000 QTrap, Framingham, MA, USA). For LCMSMS the IN.PACT Admiral sample proteins were precipitated with methanol in a 2:1 ratio to plasma. The more rapid dissolution rate of the Lutonix DCB resulted in higher drug concentrations in the sample aliquots; consequently, the Lutonix samples were first diluted 10-fold with blank plasma and then precipitated in the same manner as the IN.PACT Admiral samples. The total amount of paclitaxel released from the deployed balloon was determined by the difference between the theoretical load and the amount of material remaining on the balloon after the brief (20 second) deployment in the bovine plasma as a simulation of the intravascular matrix. The resulting data were fitted by linear regression, or a two-phase exponential association model (GraphPad Prism version 7.00 for Windows, GraphPad Software, La Jolla, California, USA).

### 2.2. In Vivo Drug PK and Tissue Response

Tissue histological responses were analyzed following treatment with IN.PACT Admiral DCB and Lutonix DCB in the porcine iliofemoral arteries on day 0 and 30 days, 60 days, and 90 days posttreatment, and tissue drug content was determined posttreatment on day 0 and 90 days. Twenty-four (24) domestic farm swine were treated and completed follow-up until termination. Vascular access was achieved via a carotid approach. A 6F or 8F guide catheter was placed into an 8F sheath and advanced under fluoroscopic guidance into the ostium of the right or left iliofemoral artery, over a 0.035 inch guide wire. The targeted vessels were evaluated via angiography prior to treatment. Test material treatments were placed using a targeted angioplasty overstretch of 1.25:1 to 1.30:1 at the device midpoint based on pretreatment online Quantitative Vascular Angiography (QVA) measurements of the target region of the peripheral artery, and the compliance chart for each test material for DCB platforms as noted on the product labeling. This amount of device overexpansion was expected to stretch the vessels without initiating extensive vessel wall injury. A single balloon was used to treat the target peripheral artery with a single, 60-second inflation and then removed. Animals planned for time 0 analysis were immediately terminated postprocedure; all remaining animals were survived for 30 days, 60 days, and 90 days posttreatment. Gross necropsy and tissue collection consisted of removal of the treated vessel specimens for bioanalytical drug analysis and histological examination.

### 2.3. Drug Content Analysis

Porcine arterial samples were received frozen on dry ice from the In-Life test site and were stored in a -80°C freezer until homogenization. Individual tissue samples were processed using a Precellys® tissue homogenizer. The frozen arterial tissue was thawed to room temperature in the original sample container, and the weight was recorded. After weighing, the buffer volume was determined by multiplying the weight by 31; e.g., 100 mg tissue will require 3100 *μ*L buffer solution (40:60 mixture of methanol and 2.5mg/ml Sodium Dodecylsulfate (SDS) in 50 mM Ammonium Acetate, pH 4) for the preparation of the homogenate. Tissue was then cut with a razor blade into pieces of approximately 1 mm^2^. The pieces, ~100-200mg, were placed in 2mL Eppendorf tubes with zirconium oxide beads. An additional 900ul of tissue buffer was then added to tubes which were placed in the Precellys for a total of 2 minutes at 5500rpm. After the homogenization, the samples were checked to see if tissue was completely homogenized, and if not, the samples were reprocessed for another cycle of 2 minutes. Once the sample was fully homogenized, it was combined with the remaining buffer. The final suspension was frozen at -80°C until analysis.

The homogenized tissue suspension was thawed in ice water, and after vigorous mixing an aliquot of 150 *μ*L was pipetted with a wide bore pipette tip into a conically shaped Eppendorf tube. Paclitaxel was extracted by the addition of 300 *μ*L methanol with 50ng/ml paclitaxel-d5 as an internal standard. Following vortexing and centrifugation, the supernatant was transferred into HPLC vials for analysis by LCMSMS. The sample analysis was performed using a HPLC method with a ballistic gradient and detection using an electrospray ion (ESI) source with multiple reactions monitoring.

For quantitation, the peak area ratio of the analyte and the internal standard were used to establish a weighted linear regression curve. For each batch, at least two standard curves with at least 8 concentrations other than zero were prepared over the range of the assay. In addition, replicates of quality control samples were prepared over the range of the assay. Each unknown sample was injected once during the analysis of a batch of samples. The dilution factor of 0.032 was applied to convert the concentration ng/mL to ng/mg for the arterial tissue samples.

Statistical comparisons of drug concentrations in tissue were determined on the day of treatment (day 0) and at 90 days posttreatment. Mean values and standard deviations were calculated using GraphPad Prism 7.0 and comparisons were conducted using a Mann-Whitney test.

### 2.4. Histopathology Analysis

While paclitaxel may inhibit cell proliferation though multiple mechanisms, the apoptotic cellular responses to high concentrations of paclitaxel are well established as relevant to smooth muscle cells (SMCs) responses in vivo and linked to arterial wall remodeling in treated segments [4]. Consequently, loss of SMCs from the arterial media of treated segments was employed as an indicator of paclitaxel activity and objectively quantified at multiple time points, up to 90 days posttreatment.

Histopathology was conducted independently at CVPath Inc., Gaithersburg, MD, USA. All vessel segments were dehydrated in a graded series of alcohols and xylenes. All segments were further segmented at ~3 to 4mm intervals and embedded in paraffin. The most proximal segment was offset to maintain longitudinal orientation. Histologic sections were then cut on a rotary microtome at 4 to 6 microns, mounted on charged slides, and stained with hematoxylin and eosin (H&E) and Movat Pentachrome connective tissue stain.

A single proximal section and single distal section from each artery closest to the mid-treated segment were selected for analysis. To assess arterial response to drug treatment, ordinal histopathological data was collected to characterize smooth muscle cell loss (Table 1). Scores were expressed as mean ± standard deviation. Wilcoxon rank sum tests were used to calculate the significance of differences between scores. A Chi-Square value of* p* ≤ 0.05 was considered statistically significant.

## 3. Results

### 3.1. Paclitaxel Dissolution

Based on analysis of the residual drug remaining on the balloon, the total paclitaxel available for solubilization was determined to be 687 ± 250 *μ*g for IN.PACT Admiral DCB and 320 ± 122 *μ*g for Lutonix DCB which is equivalent to 14% ± 5.5 and 12% ± 6.0 of the total DCB drug load, respectively; normalized dissolution values were calculated based on the total solid-phased drug load released from the balloon.

**Figure 1:**
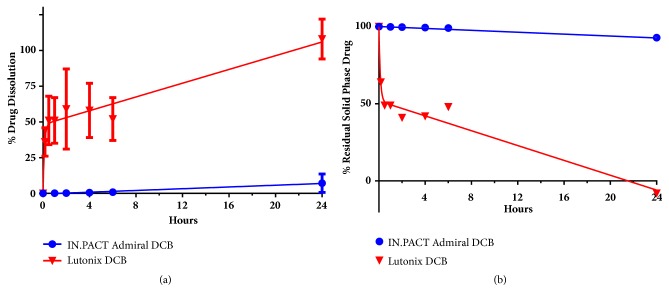
Dissolution of solid phase paclitaxel in plasma shown as nonconstrained normalized mean percentage of total solid phase drug solubilized ± SD (a) or remaining solid phase drug in suspension (b) calculated by subtraction from total normalized input drug.

The normalized dissolution data for IN.PACT Admiral test arm (Figure 1) indicates that there was linear dissolution of 2.3 *μ*g/hour or 0.3% of input drug/hour. Solubilized paclitaxel in solution at 24 hours was 53.4 ± 49.0 *μ*g which represents 7% of drug in suspension upon delivery. The normalized dissolution data for the Lutonix DCB test arm demonstrated a pronounced biphasic behavior (Figure 1) characterized by an initially rapid solubilization of drug, with an average of 151.5 ± 35.1 *μ*g of paclitaxel dissolved in 30 minutes, constituting approximately 51% of the suspended solid phase drug and an initial dissolution rate of 304 *μ*g/hour or 102% of input drug/hour. Dissolution slowed from 30 minutes to 6 hours with solubilized drug levels ranging from 50 to 60% of the solid phase drug and a secondary dissolution rate of 10.2 *μ*g/hour or 3.1% of input drug/hour was observed after 6 hours. Following 24 hours of dissolution, the amount of paclitaxel in solution was 343 ± 126 *μ*g, which constituted all the remaining solid phase paclitaxel.

### 3.2. Arterial Tissue Drug Content

**Figure 2:**
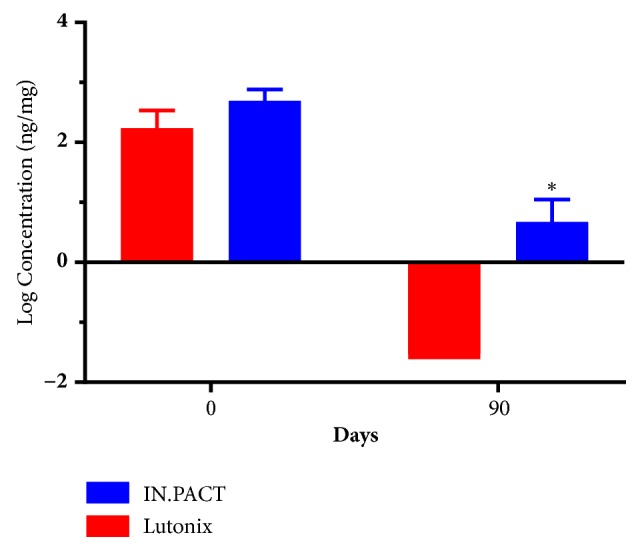
Tissue pharmacokinetics of net paclitaxel content in treated arterial segments up to 90 days posttreatment. Values are shown as log-transformed means and SD at each time point studied. *∗* indicates significant difference between arms at day 90, P= 0.0113 Mann-Whitney.

The mid-sections (approximately a third) of the treated arterial tissues were selected as representative of the entire treated segment concentrations and submitted for analysis of paclitaxel content.  Figure 2 provides the average paclitaxel concentrations in the treated tissue for each arm from 0 to 90 days. Comparison of tissue drug concentration between device arms was significant at P=0.0113 (two-tailed Mann-Whitney test).

### 3.3. Histological Response to Drug

**Figure 3:**
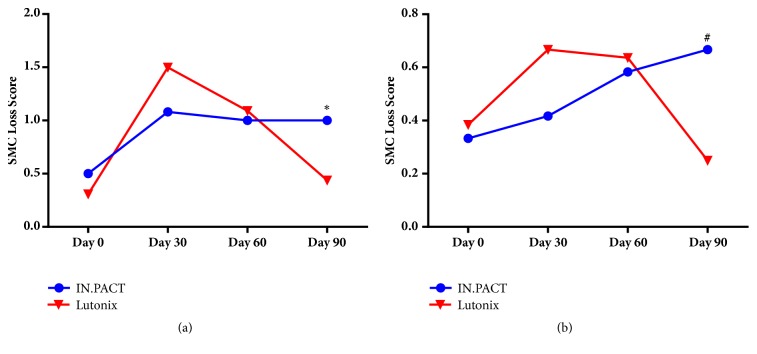
Histological scores of smooth muscle cell (SMC) loss indexed by depth (a) and circumference (b). Symbols indicate mean scores at each time point for IN.PACT Admiral (blue circles) and Lutonix (red triangles). Significant differences at 90 days by Mann-Whitney, *∗*P=0.0380, ^#^P=0.0304.

**Figure 4:**
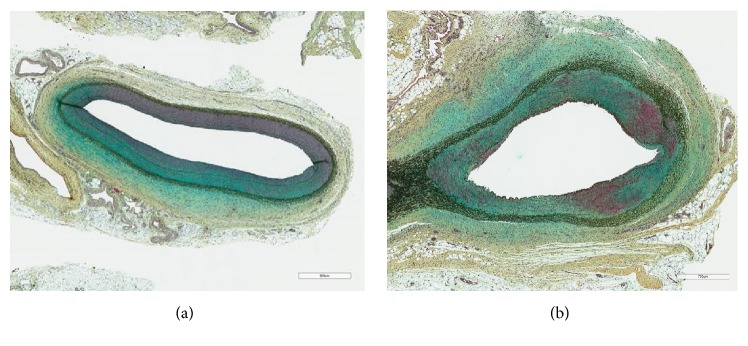
Movat pentachrome stain of arterial sections 90 days posttreatment with Lutonix DCB (polysorbate/sorbitol/paclitaxel formulation; (a)) or IN.PACT DCB (urea/paclitaxel formulation; (b)). Areas of green staining indicate zones of SMC loss and proteoglycan deposition associated with local drug activity. Bar indicates 700 *μ*m.

Medial SMC loss was employed as an indicator of paclitaxel-associated induction of apoptosis, one of established mechanisms of drug action in controlling neointimal proliferation. Two different trends of SMC loss following treatment (Figures 3 and 4) were observed with the different DCBs, indexed by both the depth and circumferential extent of involvement in the arterial media. The Lutonix DCB produced a sharp rise in SMC loss early in the posttreatment phase, followed by progressively declining scores from 30 to 90 days. In contrast, the IN.PACT Admiral DCB produced a more gradual trend in SMC loss, increasing or maintaining effect through 90 days. Although the magnitude of SMC loss was similar at most time points posttreatment with both devices, at 90 days, a significantly greater loss, both in depth (P=0.0380) and circumferential involvement (P=0.0304), was seen with IN.PACT Admiral DCB compared to the Lutonix DCB, indicating a significantly greater treatment effect at this time point.

## 4. Discussion

DCBs have been shown to be safe and efficacious in preventing restenosis following drug delivery in peripheral artery disease [5, 6] and their promise continues to drive application into additional indications. All clinically available devices utilize paclitaxel as the key API for suppressing restenosis, although with different formulations which utilize different API concentrations and various excipients [7]. However, the functional characteristics of the formulated coating, and consequently the DCB, may be argued to be dependent upon the interaction of the excipient with the drug, balloon, and application site. In the current work, two different DCBs were selected for comparison: IN.PACT Admiral DCB (3.5 *μ*g/mm^2^ paclitaxel with a urea excipient; Medtronic PLC) [8] and Lutonix DCB (2 *μ*g/mm^2^ paclitaxel with a polysorbate/sorbitol excipient; Bard PV) [9]. While both formulations facilitate release of the coating from the balloon, the interaction of the excipients with the drug may differ, owing to their chemical characteristics.

Urea is a highly hydrophilic molecule which dissolves readily in aqueous solutions as well as alcohols, although it and its aliphatic derivatives are poorly hydrotropic molecules in combination with paclitaxel, having minimal impact on drug solubility [10, 11]. Likewise, both sorbitol and its higher molecular weight derivatives, polysorbates, are highly hydrophilic [12]; however, unlike urea, there is a greater likelihood of interaction with lipophilic substances due to their chemical structure. Polysorbate is a recognized surfactant and emulsifying agent and is employed for formulating docetaxel for systemic administration in oncology applications [12]. Sorbitol is a sugar alcohol known for its hydrating properties and capacity to solubilize in both water and organic solvents. Consequently, inclusion of a polysorbate and sorbitol-based excipient into a formulation with paclitaxel may reasonably be expected to facilitate dissolution of the lipophilic drug in an aqueous medium. Comparison of these two different formulations provides an opportunity to examine the effect of rate of paclitaxel solubilization on the local pharmacological response and the potential for clearance of the drug from the treatment site.

The* in vitro* comparison of coating dissolution from the urea-based IN.PACT Admiral DCB and polysorbate/sorbitol-based Lutonix DCB revealed two strikingly different rates and manner of dissolution in plasma. IN.PACT Admiral DCB coating demonstrated a slower and constant rate of drug solubilization, suggesting that the inclusion of a urea excipient facilitates a continuous release of drug form solid to soluble phase. The Lutonix DCB showed a faster transition of half of the solid phase drug into soluble phase, suggesting that (1) inclusion of a polysorbate/sorbitol excipient accelerates solubilization of associated drug and (2) there are likely two different forms of the solid phase formulation leading to two different release rates, although both rates are greater than that seen with the urea-based formulation. Consequently, the inclusion of different excipients results in sharply contrasting functional behavior. The overall rate of drug release from a solid phase formulation has been described as having two steps, disintegration and dissolution, in which there can be two different rate limiting steps [13]. In the case of the IN.PACT Admiral DCB with urea formulation, there seems to be a single rate limiting step which is likely attributable to dissolution from solid phase to free soluble form in association with the matrix (plasma). While urea may aid in the initial disintegration of the solid phase formation due to its hydrophilic nature, the minimal hydrotropic contribution from urea [11] makes it unlikely to impact the rate limiting dissolution of drug. In the contrast, the Lutonix DCB formation results in a clearly biphasic process in which both disintegration and dissolution may contribute to the overall rapid release rate of the drug. Such a process is supported by the hydrating effect of sorbitol and the strongly hydrotropic nature of polysorbate [12], although, based on apparent sequential and discontinuous release rates of drug with time, it is unclear whether the disintegration or dissolution is the rate limiting step in drug solubilization.

In previous studies, differential rates of drug release have been attributed to the crystallinity of the solid phase formulations, leading to a differential compartmentalization of released drug [4], owing to both the association of solid phase drug with the lumen of the vessel and soluble drug with a tissue associated pool transported by convective and diffuse movement in the vessel wall. The consequences of such a differential distribution are (1) more rapid onset of drug action associated with greater quantities bioavailable drug in tissue and (2) more rapid clearance of bioavailable drug from the tissue due to normal drug clearance mechanisms than drug retained in solid phase. Both differences may be seen between the two formulations evaluated in the current studies.

Total drug content in the treated vascular tissue is similar immediately following the deployment of both DCB types. However, differential clearance of drug in tissue leads to a significant difference in total drug content at 90 days posttreatment. This difference is further accentuated by a difference in drug content (dose density) in the DCB formulations, 3.5 *μ*g/mm^2^ vs. 2 *μ*g/mm^2^. While the compartmental distribution of drug could not be directly investigated, due to study duration and remodeling of the vascular tissue with time, the difference in total drug content is consistent with previous studies [4] and suggests that slower dissolution of drug will result in a longer duration of total drug in tissue.

Histological observation of SMC loss is consistent with differential rates of bioavailable drug release. The SMC loss seen following the Lutonix DCB treatment rises rapidly at a month following treatment then declines through 90 days as the net drug content in tissue declines. The SMC loss observed with the IN.PACT Admiral DCB increases at 30 days and is maintained through 90 days at levels significantly greater than that observed for the Lutonix DCB, in alignment with significantly greater levels of drug in tissue. Since suppression of SMC proliferation through loss of proliferating populations of cells by apoptosis and inhibition of other cellular mechanisms contributes to control of vascular intima and ultimately the maintenance of vascular patency, the durability of therapeutic action could be impacted by sustained pools of bioavailable drug at the treatment site long after the therapeutic procedure. The potential for durability of clinical responses to DCB treatment highlights the importance for careful design of the coating formulation to optimize formulation for the desired duration of pharmacological response [14].

## 5. Conclusion

Differential rates of bioavailable drug release from DCBs employing different excipients can impact the duration of drug in tissue and the resulting pharmacological activity at the site of drug delivery. An excipient with a low hydrotropic potential, such as urea, will produce a slower more even dissolution of drug than an excipient with high hydrotropic potential, such as polysorbate/sorbitol. Consequently, formulation selection must be carefully considered to provide a drug release profile most suitable for the desired pharmacological effect.

## Figures and Tables

**Table 1:** Semi-quantitative analysis of pathologic changes in peripheral arteries.

	**0 (none)**	**1 (minimal)**	**2 (mild)**	**3 (moderate)**	**4 (severe)**
**MEDIAL CELL LOSS**					

**SMC Loss (Depth)**	none	Smooth muscle loss <25% of medial thickness	25-50% of medial thickness	51-75% of medial thickness	>75% of medial thickness

**SMC Loss (Circumference)**	none	<25% of circumference	25-50% of circumference	51-75% of circumference	>75% of circumference

## Data Availability

All the data regarding “in vitro paclitaxel dissolution, in vivo drug PK, arterial tissue response, and histological analysis” used to support the findings of this study are available from the corresponding author upon request.

## References

[1] Tepe G., Laird J., Schneider P. (2015). Drug-coated balloon versus standard percutaneous transluminal angioplasty for the treatment of superficial femoral and popliteal peripheral artery disease: 12-month results from the IN.PACT SFA randomized trial. *Circulation*.

[2] Loh J. P., Barbash I. M., Waksman R. (2013). The current status of drug-coated balloons in percutaneous coronary and peripheral interventions. *EuroIntervention*.

[3] National Center for Biotechnology Information. PubChem Compound Database, CID=36314. https://pubchem.ncbi.nlm.nih.gov/compound/36.

[4] Granada J. F., Stenoien M., Buszman P. P. (2014). Mechanisms of tissue uptake and retention of paclitaxel-coated balloons: impact on neointimal proliferation and healing. *Open Heart*.

[5] Scheller B., Hehrlein C., Bocksch W. (2006). Treatment of coronary in-stent restenosis with a paclitaxel-coated balloon catheter. *The New England Journal of Medicine*.

[6] Tepe G., Zeller T., Albrecht T. (2008). Local delivery of paclitaxel to inhibit restenosis during angioplasty of the leg. *The New England Journal of Medicine*.

[7] Franzone, EugenioStabile E., Trimarco B., Clavijo L. C. (2015). Peripheral Drug-Eluting Technology. *Cardiology Clinics*.

[8] IN.PACT™ Admiral Paclitaxel-eluting PTA Balloon Catheter Instructions for Use, Medtronic PLC. https://www.accessdata.fda.gov/cdrh_docs/pdf14/P140010c.pdf.

[9] Lutonix*Ⓡ* 035 Drug Coated Balloon PTA Catheter Instructions for Use, BARD. https://www.accessdata.fda.gov/cdrh_docs/pdf13/P130024d.pdf.

[10] U.S. Environmental Protection Agency Toxicological Review of Urea, IRIS Database. https://www.epa.gov/iris.

[11] Lee J., Lee S. C., Acharya G., Chang C.-J., Park K. (2003). Hydrotropic solubilization of paclitaxel: Analysis of chemical structures for hydrotropic property. *Pharmaceutical Research*.

[12] Hennenfent K. L., Govindan R. (2006). Novel formulations of taxanes: a review. Old wine in a new bottle?. *Annals of Oncology*.

[13] Gowthamarajan K., Singh S. K. (2010). Dissolution testing for poorly soluble drugs: A continuing perspective. *Dissolution Technologies*.

[14] Laird J. R., Schneider P. A., Tepe G. (2015). Durability of treatment effect using a drug-coated balloon for femoropopliteal lesions: 24-month results of IN.PACT SFA. *Journal of the American College of Cardiology*.

